# Complete Genome Sequence of NAH7-Harboring Pseudomonas putida Strain G7

**DOI:** 10.1128/mra.00394-22

**Published:** 2022-09-21

**Authors:** Paige M. Varner, Claudia K. Gunsch

**Affiliations:** a Department of Civil and Environmental Engineering, Duke University, Durham, NC, USA; University of Arizona

## Abstract

Pseudomonas putida G7 (111) is an aerobic bacterial species discovered in soil known to harbor the naphthalene-degrading NAH7 plasmid. Here, we report the genome sequence of G7. The genome was assembled by Nanopore sequencing and consisted of a chromosome of 6.4 Mbp with a G+C content of 62.13%.

## ANNOUNCEMENT

Pseudomonas putida 111 (commonly referred to as strain G7 or ATCC 17485) is a bacterium known for harboring the naphthalene-degrading plasmid NAH7. It was first isolated by W.R. Sistrom from soil enriched with naphthalene (1). P. putida G7 (hereon G7) has been studied often in regard to its motility or as a host of the well-characterized NAH7 plasmid (2–5). We report the complete genome sequence of P. putida G7 obtained by Nanopore sequencing, to better understand Pseudomonas biology and as a host to a degradative plasmid of engineering significance.

G7 was obtained from ATCC under number 17485. A single colony was picked and cultured on Luria broth (LB) medium at 30°C until reaching its exponential growth phase (OD600 of 0.5). Total genomic DNA was extracted using Qiagen’s Genomic DNA Extraction protocol with the Genomic-tip 500/G kit (Qiagen, Venlo, the Netherlands). A quality score of 2.7 for 280/260 absorbance was obtained prior to sequencing. Library preparation was performed using the Oxford Nanopore Technologies (ONT) Rapid Sequencing Kit (SQK-RAD004). Sequencing was performed on the ONT MinION using a R9.4.1 flow cell and fast base calling, and sequencing was monitored with the MinKNOW software v4.0.13 (ONT, Oxford, UK). There were a total of 227,618 postfiltered reads with an average length of 9,506 bp. Reads with a length of 10 kb or more were retained for assembly resulting in 200× coverage. Reads were assembled with Flye v2.8.1 with default parameters which includes one round of polishing (6). The assembly was further polished and circular contigs were reoriented to start at the origin of replication with Medaka model r941_min_fast_g303, v1.2.0f (ONT, Oxford, UK).

Assembly quality and completeness was assessed first by QUAST (v5.0.2) to report basic assembly statistics, such as N50. BUSCO (v4.1.4) was used to assess assembly completeness by searching for single-copy conserved orthologs using the “auto-lineage selection” parameter to select the appropriate odb10 database (7, 8). QUAST and BUSCO results were summarized with multiQC (v1.9) (9). An N50 of 6,376 kb was reported and 96.2% of BUSCOs were complete and single copy. To confirm the species sequenced and check for contamination, the contig from the assembly was queried against the blast nt database using the megablast algorithm (v2.7.1). Results showed a 99.168% identity to Pseudomonas putida KF715. Minimap2 (v2.17) was used to assess depth of coverage across the contigs by mapping reads to the polished assemblies (10). To check for mis-assemblies, each assembly was aligned to itself using minimap2 with the “asm5” preset settings for aligning genomes to one another. No mis-assemblies were found.

The complete genome of this strain is 6,375,733 bp with a G+C content of 62.13%. The NCBI Prokaryotic Genome Annotation Pipeline (PGAP) found 6,067 total genes with 5,506 being protein coding and 104 being RNA genes (11). Twelve genes were found to be involved in the chemotaxis of G7 which supports previous studies (3–5). Additionally, multiple Type IV pilus assembly proteins were identified which might aid in the conjugation/transfer of the NAH7 plasmid harbored in G7, making G7 attractive for its potential to be used in genetic bioaugmentation schemes for remediation of polycyclic aromatic hydrocarbons (12).

This is a report of the complete genome sequence of P. putida G7. This assembly will facilitate genome-wide comparison studies and development of microbiome engineering strategies for bioremediation.

### Data availability.

The chromosome sequence and annotation reported here was deposited in the National Center for Biotechnology Information (NCBI) GenBank Sequence Database under accession number CP096581. Raw, unfiltered fast5 sequences are submitted to the NCBI Sequence Resource Archive under BioProject accession number PRJNA831801.
